# Recent Advances in Polyurethane for Artificial Vascular Application

**DOI:** 10.3390/polym16243528

**Published:** 2024-12-18

**Authors:** Hua Ji, Xiaochen Shi, Hongjun Yang

**Affiliations:** 1 Winner Institute for Innovation Research, Winner Medical Co., Ltd., Wuhan 430070, China; jihua@winnermedical.com; 2 College of Materials Science and Engineering, Wuhan Textile University, Wuhan 430070, China; h_j.yang@yahoo.com; 3 Key Laboratory of Green Processing and Functional New Textile Materials of Ministry of Education, Wuhan Textile University, Wuhan 430070, China

**Keywords:** polyurethane, biostability, biocompatibility, biodegradability, artificial blood vessels

## Abstract

Artificial blood vessels made from polyurethane (PU) have been researched for many years but are not yet in clinical use. The main reason was that the PU materials are prone to degradation after contact with blood and will also cause inflammation after long-term implantation. At present, PU has made progress in biostability and biocompatibility, respectively. The PU for artificial blood vessels still requires a balance between material stability and biocompatibility to maintain its long-term stability in vivo, which needs to be further optimized. Based on the requirement of PU materials for artificial vascular applications, this paper views the development of biostable PU, bioactive PU, and bioresorbable PU. The improvement of biostable PU from the monomer structure, chemical composition, and additives are discussed to improve the long-term biostability in vivo. The surface grafting and functionalization methods of bioactive PU to reduce thrombosis and promote endothelialization for improving biocompatibility are summarized. In addition, the bioresorbable PU for tissue-engineered artificial blood vessels is discussed to balance between the degradation rate and mechanical properties. The ideal PU materials for artificial blood vessels must have good mechanical properties, stability, and biocompatibility at the same time. Finally, the application potential of PU materials in artificial vascular is prospected.

## 1. Introduction

Higher levels of consumption, overnutrition, and aging have made cardiovascular diseases the main non-communicable diseases worldwide [1]. The autologous arteries or veins are the most common alternative for peripheral or coronary vascular reconstruction [2]. However, many patients cannot undergo autologous vascular transplantation due to trauma, vascular disease, or prior surgery, which makes artificial blood vessels an important high-value consumable [3]. The use of vascular prostheses originated from around 1900. In the beginning, the material of metal, glass, or ivory was used in vascular prostheses, which caused blockage by clots and the death of patients [4,5]. In 1954, researchers found that it was possible to use synthetic materials as vascular prostheses, and polyester (PET), PU, and polytetrafluoroethylene (ePTFE) have proven to be the most viable materials for vascular surgery after a large number of studies [6]. ePTFE and PET are difficult to degrade in vivo and able to withstand high blood flow, which perform well as large-diameter artificial blood vessels (>6 mm), while their high thrombosis, intimal hyperplasia, or inflammatory reaction is not suitable for small-diameter artificial blood vessels (2 to 6 mm) [7]. As reported, the patency of ePTEF prostheses is 40–50% and 20% after being used to bypass the proximal popliteal artery for 5 years and infrapopliteal bypass for 3 years, respectively [8]. PU material has good biocompatibility, blood compatibility, and adjustable molecular structure, which attracts many researchers to study PU as small-diameter artificial blood vessel implants [4,9,10,11]. Furthermore, with the development of tissue-engineered artificial blood vessels, the hybrid materials of biodegradable materials such as poly-glycolic acid (PGA), poly-caprolactone (PCL), poly-lactic acid (PLA), and natural materials such as silk, fibrin, and chitosan are also developing [12,13,14,15].

The application of PU materials in artificial blood vessels has been studied for 60 years, not succussed in products, which is mainly related to long-term stability and biocompatibility in vivo [6,16,17]. PU are polymers with carbamate (-NHCOO-) or urea (-NHCONH-) as their main chemical structure, mainly synthesized from polyols and isocyanates [18]. The soft segment of the molecular chain composed of polyols endows the elastic material with good elasticity, toughness, and low-temperature flexibility, and the hard segment of the molecular chain composed of isocyanic acid and chain extender endows the elastomer material with high hardness, strength, and high-temperature performance [19,20]. Hard segments with strong polarity tend to aggregate, resulting in microphase separation between hard and soft segments due to thermodynamic incompatibility. Good microphase separation can make the hard phase distributed in the soft segment play the role of physical crosslinking points, thus improving the mechanical properties of elastomer materials [21]. Thus, the hard segments mainly affect the mechanical properties of artificial blood vessels. The complex blood environment contains a large proportion of water, as well as all kinds of oxygen-free radicals, proteases, acids, electrolytes, fatty tissue, and fibrous tissue around the blood vessels [22,23]. When PU artificial blood vessels contact blood for a long time, it will cause hydrolysis, oxidation, enzymolysis, calcification, and lipolysis, thus affecting their service time [19,24,25]. The degradations usually occur in the soft segment, which mainly affects the long-term stability of artificial blood vessels. In addition, the hydrophobicity of the PU surface makes it easy for non-specific protein adsorption and platelet adhesion, thereby increasing the risk of thrombosis and occlusion [26,27]. The ideal artificial blood vessel should match the original blood vessel in terms of compliance, size, hemodynamic factors, and antithrombotic and anti-inflammatory properties and promote rapid adhesion of endothelial cells [28].

At present, it has been reported that large-diameter artificial blood vessel products manufactured by commercial biomedical polycarbonate polyurethane (PCU) have excellent blood compatibility and biocompatibility, a high endothelialization rate, and 100% patency [4]. This brings hope for the realization of PU materials as artificial blood vessel products. Due to the degradation and inflammatory reaction of PU in vivo, many researchers have aimed to change the chemical structure of PU to adapt to the internal environment. However, few reports systematically summarize the application of different PU materials in artificial blood vessels. This paper will focus on the chemical composition of PU and summarize the different types of PU materials and the improvement in the application of artificial blood vessels in recent years to promote the application process of polyurethane artificial blood vessel products.

## 2. PU Artificial Blood Vessels

**Figure 1:**
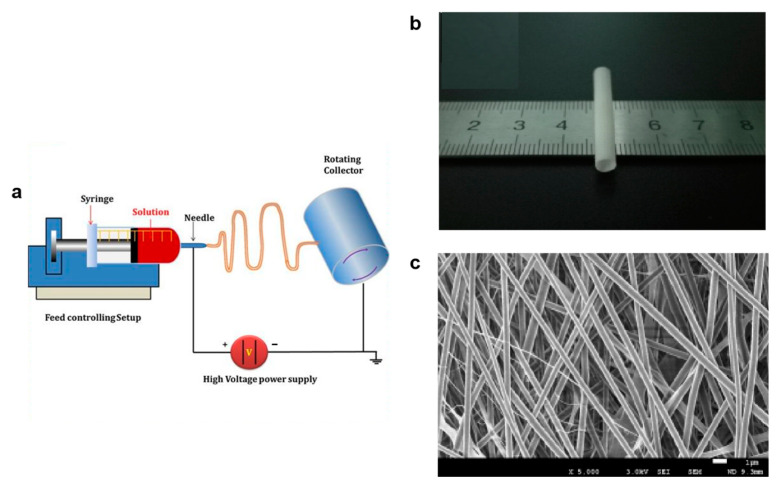
(**a**) The electrospinning technique [36]. Copyright 2017, Elsevier Ltd. (**b**) the PU artificial blood vessels made by electrospinning, (**c**) the nanofibers of PU artificial blood vessels [37]. Copyright 2017, Elsevier Ltd.

PU artificial blood vessels are mainly used to temporarily or permanently replace the defective arteries or veins of patients, can be used as a countercurrent channel when arteries are blocked, or as an alternative tube for arteriovenous transplantation in kidney patients undergoing hemodialysis [29,30]. PU vascular grafts are usually prepared using electrospinning technology as shown in Figure 1; the polymer solution in the syringe forms a Taylor cone at the tip of the nozzle under the high-voltage current, and the charged jet carries out a whipping movement under the electric field; the solvent evaporates and forms fibers on the collector [31,32,33]. The produces fiber diameters that can be controlled at the nanometer and micron levels by adjusting the viscosity and flow rate of polymer solution, applied voltage, spinning distance, and ambient condition, and the diameter of vascular graft can be controlled by the size of the collector [34,35]. In addition, the vascular graft can be modified and designed with multilayer structures by the spinning solution to improve its application.

**Figure 2:**
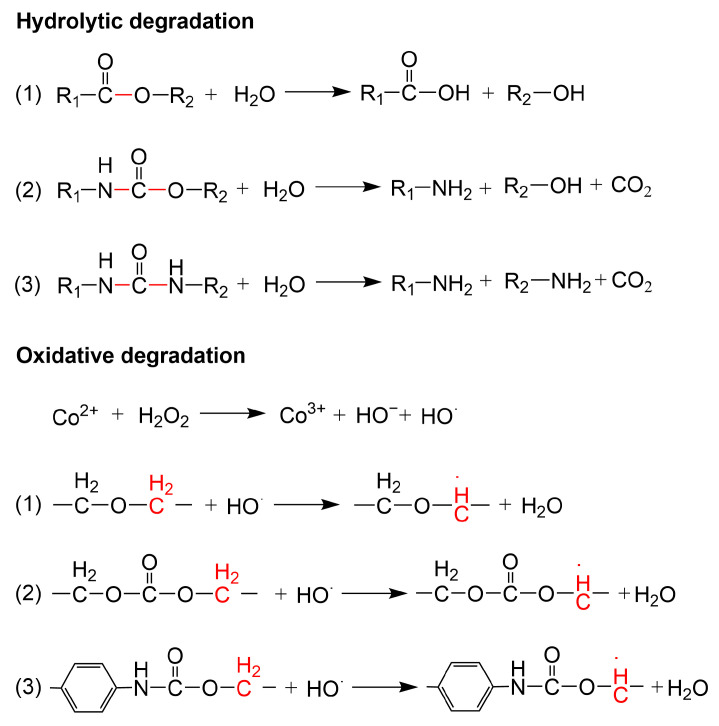
The hydrolysis and oxidative degradation mechanism of PU in vivo.

Zhang et al. [38,39] studied the behavior of artificial blood vessels, Vascugraf^®^, made of PCU materials in vivo, and the results showed that a small number of microfibers appeared cracked after one month when blood vessels were implanted in dogs. After 358 days of implantation in humans, although the material remained stable, a small number of microfiber cracks appeared. These cracks were mainly caused by the degradation of PU in vivo. The α-CH_2_- near the ester and ether bond in the soft segment of PU is a weak bond that is easy to degrade, resulting in bond breakage [40]. The H^+^, enzymes (oxidase and hydrolase), and oxidizer (O_2_^−^, H_2_O_2_,^·^OH) which were released by inflammatory cells, acidic substances (such as fatty acids), and substances in body fluids (Ca^2+^, phospholipids, cholesterol), which constitute the degradation environment of PU in vivo, resulting in the hydrolysis and oxidative degradation of PU as shown in Figure 2 [41,42,43]. In addition, the aggregation of platelets and the adsorption of proteins on the surface of the PU are easy to cause thrombosis and reduce the patency of artificial blood vessels after long-term implantation [44]. Gu et al. [4] prepared a three-layer artificial blood vessel composed of PCU/PET; the surface of the artificial blood vessel lumen was covered with different degrees of endothelium after implantation in the thoracic aorta of the sheep for 24 weeks, and the average endothelialization rate was 69.44%, with 100% patency. Although the rapid blood flow rate in the PU large-diameter artificial vessels makes it difficult to cause thrombus, its long-term stability remains to be investigated.

To meet the implantation requirements, it is necessary to control the toxic behavior of PU in vivo to ensure its safety, and biological stability and excellent mechanical properties are also necessary. The ideal PU material for artificial blood vessels should have the following characteristics:

(1) Good biocompatibility: It will not cause serious immune rejection, inflammatory reaction, or tissue damage after implantation and has good anticoagulant properties, reducing the adhesion and aggregation of platelets. The surface of the material is conducive to the adhesion, growth, and proliferation of endothelial cells so that artificial blood vessels can gradually fuse with autologous blood vessels to form a stable vascular structure [45,46];
(2) Good mechanical properties: Artificial blood vessels need to be strong enough to withstand the pressure generated by blood flow and the extrusion of surrounding tissues and have elasticity close to natural blood vessels and good compliance to ensure the smooth flow of blood. The average burst pressures of the native saphenous vein (SV) and internal mammary artery (IMA) were 1599 ± 877 mmHg and 3196 ± 1264 mmHg, respectively, and the compliance of SV was 11.5 ± 3.9%/100 mmHg, the suture retention strength of IMA was 138 ± 50 gmf [47];
(3) Good biostability: The material should have good chemical stability, will not cause degradation, crosslinking, oxidation, and other chemical reactions, can resist the acid-base environment, the action of enzymes, and other chemical substances erosion [48,49].

## 3. Chemical Composition of PU

**Figure 3:**
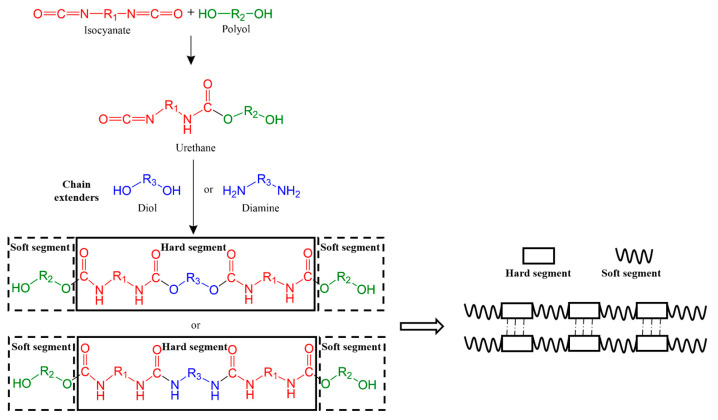
The chemical reaction of PU synthesis and representation of soft segment and hard segment.

PU is composed of polyols, isocyanates, and chain extenders. Polyols are oligomeric macromonomers of low molecular weight. Chain extenders are typically small molecules carrying hydroxyl or amine groups. Isocyanates are components of low molecular weight, capable of reacting with polyols or chain extenders to form segmented polyurethane structures [50]. The influence of monomer structure on the main properties of PU material is shown in Table 1. The synthesis of PU can be carried out via one-step or two-step methods. In the one-step method, different proportions of polyols, isocyanates, and chain extenders are mixed and reacted together, which is simple but difficult to control the chemical structure of PU. The two-step method makes it easier to regulate the performance of PU [51]. The two-step synthesis process is shown in Figure 3: First, polyols react with excess isocyanates to form urethane, commonly known as “prepolymer”; then the residual isocyanate functional groups react with the added chain extenders to form high molecular weight polymers. These three monomers are the basic compounds of PU, and their chemical structures have a significant impact on the final performance of PU [52,53,54]. Therefore, understanding their functional roles is important for designing PU materials that meet specific requirements.

### 3.1. Polyols

Polyols are mainly divided into polyether polyols and polyester polyols; the branch chain, length, and structure will affect the properties of PU materials., Common polyols are shown in Table 2. Jena et al. [55] used hyperbranched polyester polyol for the synthesis of PU, which has better thermal stability and can enhance the heat resistance of PU materials. Moreover, the addition of hyperbranched polyester polyol can also improve the hydrogen bonding and mechanical properties of PU [56]. Šebenik et al. [57] studied the effect of different molecular weights and contents of polypropylene glycol on PU, and the results showed that high molecular weight polyol increased the flexibility of PU, and increasing the content of high molecular weight soft segments would reduce its glass transition temperature. The degree of soft segment crystallinity and microphase separation was increased with the increasing molecular weight of polyol, which led to an increase in tensile strength and the elongation at the break of PU [58]. Due to the presence of ester groups in polyester polyol, it is easy to cause the hydrolysis of PU in vivo, which leads to the deterioration of its mechanical properties [59]. The backbone of polyether polyols consists of the ether group and hydroxyl group, and the ether group can only be cleaved in a strongly acidic environment, which has higher hydrolytic stability. However, ether bonds are prone to oxidation, and the effect of oxidation and mechanical prestress causes surface cracking and fracture of hydrolyzed and stable PU in vivo [60]. Therefore, many researchers have made PU materials stable by changing the structure of polyols, making them hard to degrade in vivo, such as polyalkyl polyols, polydimethylsiloxane polyols, and polycarbonate polyols [61,62,63]. Furthermore, some polyols, such as PCL, PEG, and PLA, are nontoxic and bioabsorbable, which can increase the biocompatibility of PU in vivo.

### 3.2. Isocyanates

Isocyanates can be aliphatic [64,65], cycloaliphatic [66,67], and aromatic [68,69], contain two or more -N=C=O groups in the molecular structure and are important compounds in PU synthesis. Common polyols are shown in Table 3. Multifunctional isocyanates react to form a network or crosslink. Isocyanates and chain extenders form chain segments that are more rigid than polyol due to strong intermolecular interactions, which are usually glassy at room temperature and are called hard chain segments. The reactivity of isocyanates is governed by the positive charge of the carbon atom and is susceptible to attack by nucleophiles, oxygen, and electrophiles. Thus, aromatic isocyanates are more active than aliphatic or cycloaliphatic isocyanates. Juhasz et al. [70] studied the reaction kinetics of hexamethylene diisocyanate (HDI), 4,4′-dicyclohexylmethane diisocyanate (HMDI), and isoferone diisocyanate (IPDI) to synthesize PU and found that the reaction activity of diisocyanate was HDI > IPDI > HMDI.

Under the same soft segment structure, the mechanical properties of PU mainly depend on the content and chemical structure of the hard segment [71]. Segmented polyurethane materials with symmetrical structural isocyanates (MDI, HMDI, and HDI) generated have high hard segment crystallite and exhibit high Young’s modulus and hardness [72,73]. PU synthesized from aliphatic isocyanates exhibited poorer mechanical properties and stability in vivo compared to aromatic isocyanates. This is because the delocalization of π electrons on the aromatic ring prevents the rotation of the molecule, giving it a rather rigid molecular structure, while the high flexibility of aliphatic diisocyanates is detrimental to the ordering and cohesion of the hard segments. However, aromatic diisocyanates degrade in vivo to form aromatic amines that are more toxic than aliphatic ones, and the resulting polymers are exposed to natural light to form quinone chromophores, resulting in the yellow color of PU [73,74]. Recent studies have also used cyclic carbonate-ammonolysis to obtain non-isocyanate PU to avoid its toxicity, but the kinetics of the synthesis process is slow, and the forming PU has a lower molecular weight [75]. Also, biobased isocyanate, which is directly from biomass, is an alternative to replace fossil-based isocyanates to enhance their sustainability [74].

### 3.3. Chain Extender

Chain extenders are usually low molecular weight (<400) active hydrogen-containing compounds, most of which are bifunctional compounds, typically including ethylene glycol, diamine, and amino alcohols, as shown in Table 4 [76,77,78]. The prepolymer produced by the reaction of polyol and diisocyanate is a soft rubber with poor mechanical strength. The chain growth chain organization and sequence distribution in the copolymer can be guided by the addition of a chain extender. These extensions, composed of chain extenders and diisocyanates, act as both filler particles and physical cross-linking sites to increase mechanical strength [79]. As a part of the hard segment, the mechanical properties of PU are related to the content of the chain extender and chemical structure. When linear diol is used as the chain extender to obtain thermoplastic polyurethane (TPU) that can be processed hot, and diamine is used as the chain extender to obtain the structure of urethane, the urea group provides additional N-H function in the polymer chain and increases the degree of hydrogen bonding between ethyl carbamic ester, thereby increasing the hard segment cohesion density and increasing the strength of polyurethane, which is usually thermoset material, which is not favorable for processing [80]. Qin et al. [77] studied the effects of 1, 4-butanediol (BDO), aminoethanol (MEA), and ethylene glycol (EG) as chain extenders on the properties of TPU, and the results showed that BDO and EO with symmetrical structure had good phase and mechanical properties. In addition, other studies have shown that the PU modulus increases with the increase of the number of methylene chain extenders, while the PU modulus decreases when there are six methylene groups, forming a more ordered hard segment microdomain [81].

## 4. Biostable PU for Artificial Blood Vessels

**Figure 4:**
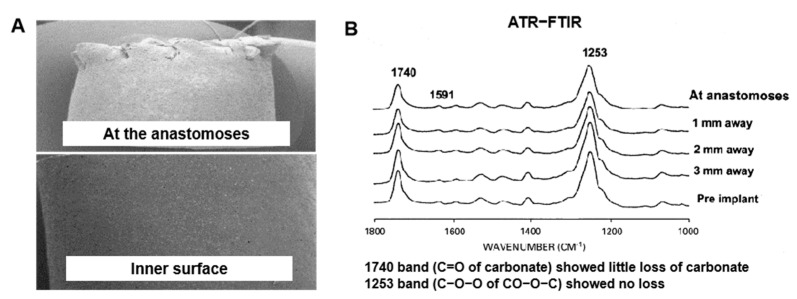
(**A**) ESEM pictures of Myolink™ graft post-implant, (**B**) the ATR-FTIR result of Myolink™ graft, pre-implantation (control) at distal anastomoses and 1, 2, and 3 mm away from anastomoses post-implantation [87]. Copyright 2003, Elsevier Ltd.

The ester bond (-COO-) in the soft part of PU is easily hydrolyzed, but the hard part is relatively stable and not easily biodegraded [82]. In addition, hydrolysis of ester bonds can easily cause enzymatic hydrolysis of polyester polyurethane [83,84]. Poly(ether-urethane)s (PEtUs) do not contain ester bonds in their structure, and the hydrolytic break bond mainly occurs in carbamate. Because its degradation rate is one order of magnitude lower than that of ester bonds, it has good hydrolysis stability compared with polyester polyurethane [85]. Therefore, the biostable PUs commonly used on the market are mostly PEtUs and PCUs, as shown in Table 5. Gostev et al. [86] studied the biostability of Tecoflex EG-80A and Pellethane 2363-80A as the basic polymers of electrospinning blood vessels. Pellethane 2363-80A was stable for at least 6 months after implantation. The average molecular weight of Tecoflex EG-80A had a certain change after implantation for 3 months. The electrophilicity of PCU adjacent to carbonate stabilizes the chain free radicals with a lower chain-breaking rate, increases the life of the free radicals, and makes them have stronger biological stability [43]. Seifalian et al. [87] implanted the vascular graft “MyoLink™” made of PCU material synthesized by HMDI, PHECD, and ED according to a molar ratio of 2:1:0.97 into beagle dogs; the radial tensile strength was 1.48 ± 0.07 N/mm after being implanted for 3 years, and the compliance was only decreased 6%. Also, the result of attenuated total reflectance-Fourier transform infrared spectroscopy (ATR-FTIR) of pre- and post- did not show the chemical breakdown; the environmental scanning electron microscopy (ESEM) and gel permeation chromatography (GPC) also showed no signal of degradation (Figure 4). Chernonosova et al. [88] used Carbothane™ 3575A to prepare artificial vascular. The stability of scaffolds was tested by SEM and mechanically after being incubated in phosphate buffer for 1 month at 37 °C and 5% CO_2_, and the incubation led to an increase in fiber diameter and tensile strength, and the in vivo study showed it has excellent biocompatibility compared with ePTFE.

**Figure 5:**
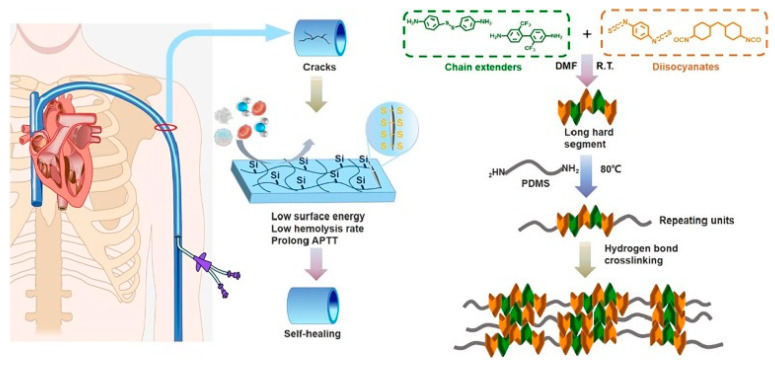
The method of PU synthesis with self-healing potential in the blood environment [99]. Copyright 2023, Elsevier Ltd.

Polydimethylsiloxane (PDMS) and macromolecular fluorocarbons have low surface energy; modification of PU ends with these compounds can increase its biological stability, and enrichment on the PU surface can hinder material degradation [94,95]. Nguyen et al. [91] studied the surface characteristics of end-modified PU materials Biospan and CarboSil, confirming that these two materials had good blood compatibility and could be used in cardiovascular devices. Kim et al. [96] treated PDMS-modified PU in lipase and 30% H_2_O_2_ for 8 weeks, and the change of mass was tested to evaluate the degradation, with no obvious weight change showing good biological stability. However, the softness of the high content of PDMS in bone chains and weak intermolecular forces will affect the mechanical properties of PU [97]. Tang et al. [98] used PDMS end-group-modified PTMEG as a polyol, MDI as a hard segment, and fluorodiol (FDO) and BDO as a chain extender to synthesize silicon-containing fluorinated polyurethane with low PDMS content. After 6 months of the implant in mice, there was no obvious degradation on the surface of the material, and the soft segment chain crack was small. Yan et al. [99] used fluorinated 2,2′-bis (trifluoromethyl) benzidine (TFMB) and 4-aminophenyl disulfide (APS) as chain extenders to improve the hydrophobicity and self-healing properties of the hard segment and used diisothiocyanate (PDITC) and HMDI as mixed diisocyanate. The long hard segment has a highly stable hydrogen bond, and PDMS is a soft segment, so the obtained long hard segment polyurethane has the characteristics of self-healing in the blood (Figure 5).

Polyhedral oligosiloxane (POSS) nanoparticles can be dissolved in a polymer matrix without reaction, are non-toxic and have good biocompatibility, and can also be used as cross-linking agents, grafted molecules, or the phase separation ability of network nodes to change material porosity [100,101]. Lewicki et al. [102] studied the degradation performance of POSS-modified PU, and the results showed that a low level of POSS substitution (<10 wt. %) reduced the yield of volatile degradation products and increased the pyrolysis polymerization temperature of PU. POSS-PCU is composed of POSS and PCU with excellent antithrombotic and mechanical properties, which can be a new type of vascular bypass graft material to maintain its machine strength while maintaining its flexibility [103]. Zakeri et al. [104] used electrospun POSS-PCU to prepare small-diameter vascular grafts, which were modified by polyacrylic acid (PAAs) grafting and soluble protein and showed good blood compatibility and angiogenesis behavior.

In addition, the stability of PU materials in vivo can be improved by adjusting the chemical composition and hard segment ratio of PU. Ramezani et al. [105] synthesized PU using HDI, polypropylene glycol (PPG), and triethylene glycol (TEG). The degradation rate of PU with high hard segment content was significantly reduced by the accelerated degradation tests in vitro, and the material could achieve adjustable mechanical properties at higher than body temperature. Zhen et al. [106] used PTMEG as the soft segment and MDI, 1, 3, 5-tri (6-isocyanohexyl) biuret (HTI), and tri (hydroxymethyl) propane (TMP) as the hard segment to synthesize biostable crosslinked PU. Through accelerated degradation with 30% H_2_O_2_ in vitro and examined using electron spectroscopy for chemical analysis (ESCA), the results showed that part of etherine was converted to carbonyl through an oxidation reaction. It has more stable properties than Pellethane and has high levels of angiogenesis and cellularity and less inflammatory response after implantation in mice for 3 weeks, which is an ideal material for promoting healing blood vessel transplantation and in situ vascular engineering.

## 5. Bioactive PU for Artificial Blood Vessels

**Figure 6:**
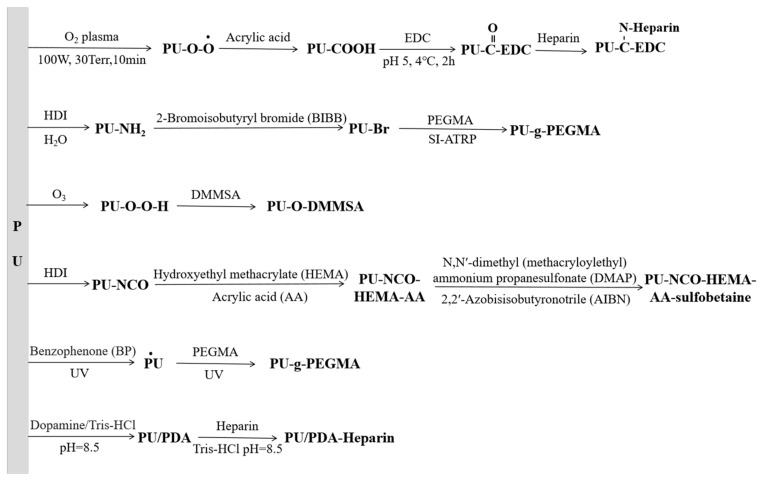
The common methods of PU surface modification [107,112,120,121,122,123].

The hydrophobicity of the PU surface makes it easy for non-specific protein adsorption and platelet adhesion. To make the polymer surface resistant to non-specific protein adhesion and enhance biocompatibility, many researchers modify PU grafts by using hydrophilic and functional substances to improve their biocompatibility in vivo. The common surface modifiers are shown in Table 6. The modifier is generally grafted directly on the surface of PU or in reactants. Common surface grafting methods are shown in Figure 6.

**Table 6:** The common surface modifier of PU.

Type	Modifier	Methods	Function	Refs.
Polysaccharides	Heparin	Oxygen plasma	Antithrombotic	[107]
	Chitosan	Oxygen plasma	*Antibacterial*, promote wound healing, reduce blood cholesterol	[108]
	Hyaluronic acid (HA)	Dopamine adhesives-assisted attachment	Antithrombotic	[109]
Fatty acid	Conjugated linoleic acid (CLA)	Oxygen plasma	Antithrombotic	[110]
Hydrophilic polymer	Poly(ethylene glycol) methacrylate (PEGMA)	Surface-initiated atom transfer radical polymerization (SI-ATRP)	Hydrophilicity	[111]
Zwitterionic polymer	Sulfobetaine	Surface oxidation	Hydrophilicity and antifouling	[112]
	Phosphobetaine	Isocyanate groups		[113]
	Carboxylbetaine	Isocyanate groups		[114]
Peptides	Arg-Gly-Asp (RGD)	Tyrosinase-mediated	Enhance endothelial cells (ECs) adhesion	[115]
	Arg-Glu-Asp-Val (REDV)	Thiol–ene reaction	Selectively adsorb and proliferate ECs	[116]
	Tyr-Lle-Gly-Ser-Arg (YIGSR)	Isocyanate groups	Enhance ECs migration and adhesion	[117]
Gasotransmitter	NO		Antithrombotic	[118]
	H_2_S		Anti-inflammatory, promoting angiogenesis	[119]

**Figure 7:**
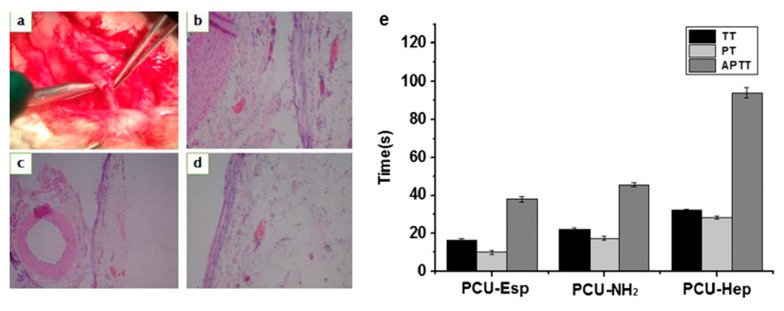
(**a**) Observation of the outside surface of PCU-Hep artificial blood vessels, (**b**–**d**) the HEparaffin section of PCU-Hep artificial blood vessels, (**e**) the coagulation test of PCU-Hep artificial blood vessels [37]. Copyright 2017, Elsevier Ltd.

Heparin is a biological anticoagulant that can enhance the activity of antithrombin III (AT-III) and inhibit the activity of coagulation factors IIa, IXa, Xa, XIa, and XIIa. After heparin combines with AT-III, it causes the conformational change of AT-III, which makes its active center more likely to bind with coagulation factors and increases the extinguishing speed of coagulation factors by about 1000 times, thus inhibiting the coagulation process and reducing the formation of fibrin, to play an antithrombotic role to reduce the occurrence of thrombosis [124,125,126]. Qiu et al. [127] explored three different grafting methods of heparin and found that the combination of plasma treatment with the introduction of amine functional groups and terminal immobilization was the most effective way to modify the graft surface of heparin. However, the etching effect of plasma will cause the mechanical properties of polyurethane to decrease with the increase of treatment time [128]. Aksoy et al. [107] used O_2_ plasma technology to activate the PU surface to produce peroxide active groups, which further reacted with acrylic acid. Then, in turn, immersed in N′-3-(dimethylaminoproply)-N-ethylcarbodiimidehydrochloride (EDC) and heparin solution preparation of the functional groups grafted heparinization polyurethane, the results showed that its surface could reduce red blood cell adhesion and had strong antithrombotic activity through a whole blood cell adhesion study. Li et al. [37] proved that the PU artificial blood vessel grafted with heparin had little effect on its mechanical properties, and the good blood compatibility and tissue compatibility were proved through blood evaluation and implantation experiments in vivo, which met the requirements of artificial blood vessels (Figure 7).

Polyethylene glycol (PEG) has high surface mobility under physiological conditions due to its hydrophilicity, and it has good resistance to plasma protein adsorption and platelet adhesion [129,130,131]. However, the graft density of PEG on the PU surface is relatively low because of the lack of surface function. The preparation of PEG methacrylate (PEGMA) on the PU surface by sulfhydryl reaction can improve the graft density of PEG [132]. Wang et al. [133] used N,N′-methylenebisacrylamide (MBAm) as a crosslinker and benzophenone (BP) as a photoinitiator. PU/PEGMA electrospun composite nanofibers were prepared by the electrospinning process. Cell proliferation tests showed that a suitable hydrophilic surface was beneficial to improve the adhesion and proliferation ability of human umbilical vein endothelial cells (HUVECs).

Zwitterionic polymers have both positive ions and anions in their chains; they can form a tightly bound water layer at the head group through hydration induced by electrostatic and hydrogen bonds in water, reducing protein adsorption and platelet adhesion [134,135,136]. Yuan et al. [112] activated the PU surface with ozone and modified the PU surface by grafting thiobetaine N, N-Dimethyl-n-methacryloxyethyl-N-(3-sulfopropyl) ammonium (DMMSA); the PU blood vessel patch showed no platelet adhesion phenomenon, and it had good anticoagulation by vitro test. Wang et al. [114] synthesized PU using carboxylbetaine triol as a chain extender; the hydrolysis of the triol segment at the interface produced zwitterionic functional groups, and the carboxylbetaine (CB) content in the polymer could be adjusted by changing the molar ratio of the soft segment and the hard segment. Crago et al. [137] grafted zwitterionic polymers onto commercial PU using a plasma method, which can reduce the fibrinogen adsorption and thrombosis by about 9 times and 75%.

Polypeptides are the main functional amino acid sequences found in the extracellular matrix, which can interact with abundant carbonyl hydrogen bonds on the PU surface and improve the hydrophilicity of the PU surface to facilitate the adhesion, growth, and proliferation of vascular ECs [138,139,140]. Choi et al. [141] first grafted PEG on the PU surface; after amination, the terminal isocyanate coupled RGD peptide to the amino end of the PEG chain, and the modified PU enhanced HUVEC activity and promoted the proliferation of HUVEC. Ding et al. [142] prepared isocyanate-coated polyurethane by organic catalytic polycondensation and coupled REDV peptide to the PU surface through sulfhydryl reaction to form the PU/REDV peptide coupling compound, which could selectively attach to HUVECs, inhibit the adhesion and proliferation of human umbilical artery smooth muscle cells (HUASMC), and reduce the adhesion of platelets.

**Figure 8:**
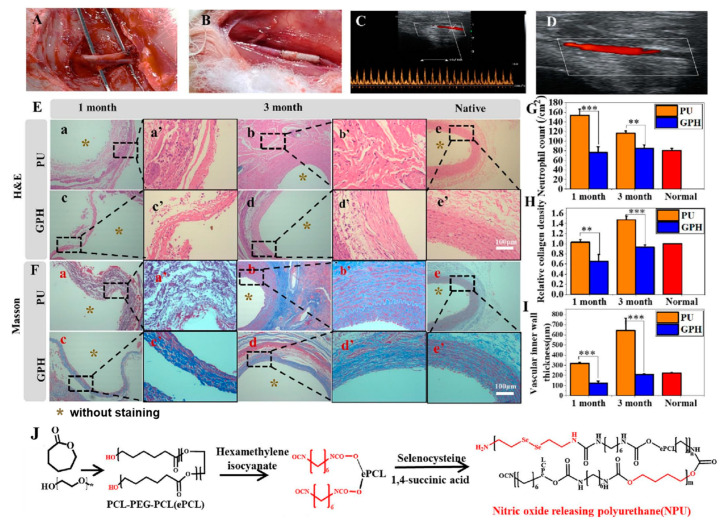
(**A**) and (**B**) The images of the carotid artery sites of rabbits before and after PU blood vessel implantation, (**C**) and (**D**) the ultrasound images of PU blood vessels after implantation for 3 months, (**E**) and (**F**) the H and E and Masson staining of different groups of PU blood vessels, respectively, (**G**) the neutrophil count of different groups of PU blood vessels, (**H**) the collagen density of different groups of blood vessels, (**I**) the vascular inner wall thickness of different groups of blood vessels, (**J**) the synthesis method of PU material with NO release function used for the blood vessels. The data are shown as the means ± S.D. from three independent experiments. ** *p* < 0.01, *** *p* < 0.001 indicates significant differences between the indicated columns [145]. Copyright 2024, Elsevier Ltd.

NO is a natural medium for vascular homeostasis. It is synthesized by L-arginine through nitric oxide synthase (NOS). NO activates guanylate cyclase in endothelial cells and catalyzes the conversion of guanosine triphosphate (GTP) to cyclic guanosine phosphates (cGMP), which dilates smooth muscle and thus dilates blood vessels. In addition, NO can also reduce the vasoconstricting substances produced by endothelial cells (such as endothelin-1), inhibit platelet aggregation and adhesion, reduce the damage of inflammation to endothelial cells, and promote the repair and regeneration of endothelial cells [143]. The rate of release into the blood is usually 0.5–4 × 10^−10^ mol cm^−2^ min^−1^ [93,144]. NO release can be achieved by loading a nitrogen source or a catalytic site into the PU molecular structure. Xiang et al. [145] introduced selenium covalent bonds into the segment structure of PU and used PCL-PEG-PCL block copolymer polyols, HDI, selenocysteine dihydrochloride, and BDO as hard segments to synthesize PU to promote the release of NO in the blood (Figure 8). The artificial blood vessels gradually degraded after implantation in rabbits for 3 months, and ECs proliferated in blood vessels, preventing inflammation and thrombosis formation, achieving long-term patency. Ji et al. [146] first grafted a polydopamine (PDA) layer on the PU surface; the prepared poly (ethyene glycol)-Heparin-Selenocystamine (PEG-Hep-SeCA) is introduced into the PDA-modified PU surface to construct a bioactive layer that can catalyze NO release. In the case of NO production, the blood compatibility and ECs growth of the PU film were significantly improved.

Similar to NO, hydrogen sulfide (H_2_S), as a third gas transmitter, plays an important role in the cardiovascular system by relaxing blood vessels, promoting ECs proliferation and migration, anti-oxidative stress, and anti-inflammatory effects [119]. Han et al. [147] synthesized a biological macromolecule H_2_S donor based on keratin-thiobenzoic acid coupling (KTC) through mercapto-disulfide exchange reaction and fixed the biological macromolecule in PU nanofibers through electrospinning technology, which can accelerate the formation of granulation tissue and promote collagen deposition and angiogenesis. Although H_2_S and NO have different functions on blood vessels, many researchers have reported that the co-release of NO and H_2_S can further accelerate angiogenesis [148,149,150,151]. Li et al. [148] designed and prepared a double-layer vascular graft with NO and H2S release ability for the first time. The vascular graft maintained patency without calcification after 6 months of implantation in the rat abdominal aorta and could promote rapid endothelialization and alleviate neointimal hyperplasia without obvious injury, inflammation, and thrombosis.

In addition, artificial blood vessels prepared by electrospinning by mixing PU with natural active materials can also improve their biocompatibility. Riboldi et al. [152] used fibroin protein mixed with PU to prepare vascular grafts, which had 100% patency and safety during 90 days of implantation between the common carotid artery and the external vein of sheep. Maleki et al. [153] prepared the anticoagulant inner layer of blood vessels by using the PU mixture grafted with fibroin protein and heparin and crosslinked gelatin and chitosan to form the outer layer to prepare blood vessels. The outer layer provided vitality for vascular smooth muscle cells (SCMs) and made blood vessels have both mechanical properties, biocompatibility, and blood compatibility.

## 6. Bioresorbable PU for Artificial Blood Vessels

Bioresorbable PU is usually used as a tissue engineering vascular scaffold, which needs to have good mechanical properties, biocompatibility, and a controllable degradation rate to match material degradation with angiogenesis. The bioresorbable PU soft segment is mostly polyester polyols with good biocompatibility. Including PLA [154,155], PGA [156], PCL [157,158,159], PEG [160,161], poly-oxyethylene (PEO) [162], and poly-lactic acid-glycolic acid copolymer (PLGA) [163,164], etc., can be safely degraded in vivo. The degradation rate of PU can be controlled by adjusting the molecular weight and structure of polyols. Zhu et al. [165] used different isocyanates, HDI and LDI, different polyols, PCL and PEG600, and putrescine as chain extenders to synthesize different degradable PUs. The results showed that PEG600 promoted the degradation of PU, and the degradation rate increased from 18% to 70% in 24 weeks, but the maximum stress was 3.8 Mpa, which was significantly smaller than PCL-based PU of 5.9 Mpa.

**Figure 9:**
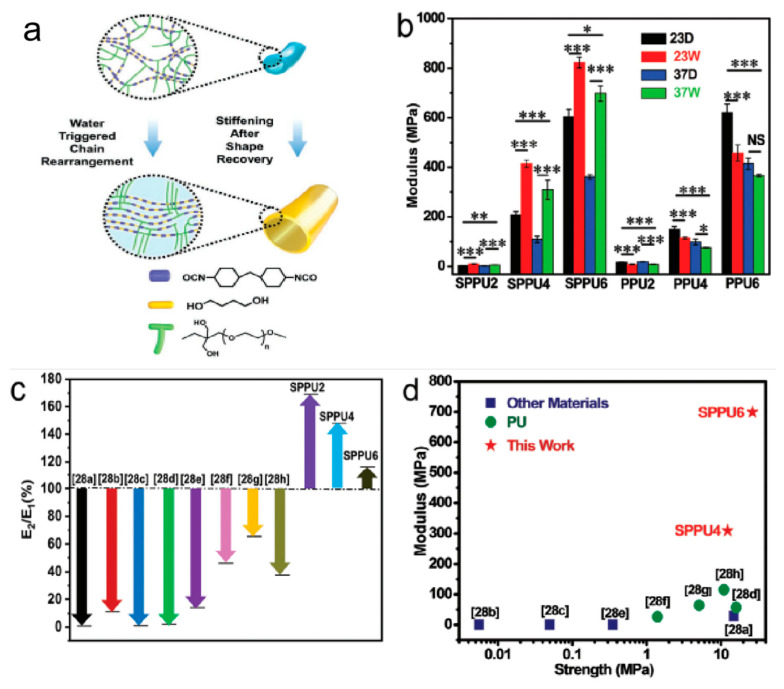
(**a**)Water-triggered stiffening of shape-memory polyurethane in the shape-recovery process, (**b**) the modulus of different PU at 23 and 37 °C, both in dry and hydrated conditions, (**c**) the modulus change, and (**d**) Young’s modulus and tensile strength of PU in this study compared to the previously reported thermally and water-triggered shape-memory materials. Results are presented as mean ± standard deviation, n ≥ 3. * *p* < 0.05, ** *p* < 0.01, *** *p* < 0.001, NS means not significance [166]. Copyright 2022, Wiley Ltd.

The rapidly degrading polymer underwater is not conducive to the control of PU mechanical properties. However, the structure can be protected by hydration-induced stiffening, and the amphiphilic polymer can be imbued with stronger underwater mechanical properties [166,167]. Liu et al. [166] designed a shape-memory polyurethane (SMPU) composed of only a hard segment on the main chain with trimethylolpropane poly(ethylene glycol) monomethyl ether (YN-120) or PEG as a soft segment; the modulus of wet PU was significantly higher than that of the dry PU due to water triggering the segment rearrangement to enhance the degree of microphase separation to achieve effective stress transfer, and the change of modulus was affected by the monomer ratios, the structure of polyethylene glycol, and temperature (Figure 9).

**Figure 10:**
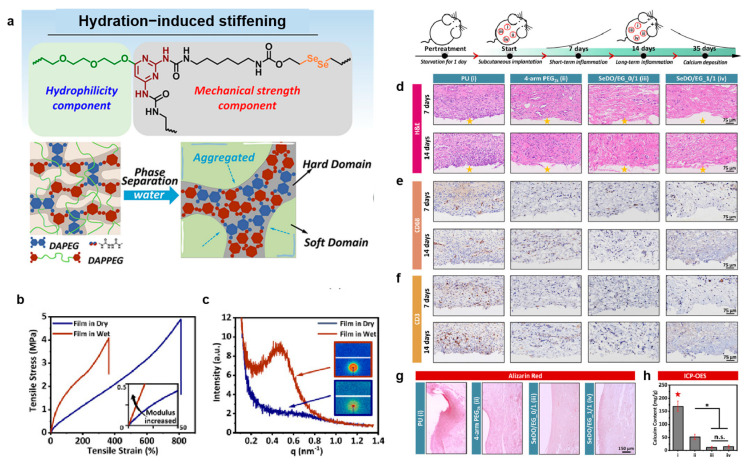
(**a**) The design of the PU elastomers, (**b**) the stress-strain and (**c**) small-angle X-ray scattering curves of DAPPEG3k/DAPEG_1/1 in wet and dry conditions, (**d**) the hematoxylin and eosin (H and E), (**e**) D68, and (**f**) CD3 images of different samples at day 7 and day 14 after implantation, (**g**) the alizarin red staining of calcium depositions, (**h**) quantitative statistics of the calcium contents detected by ICP-OES. The data were presented as mean ± SD with n = 4. * *p* < 0.05, n.s. means not significant, the dull red pentacle Indicates that this group was significantly higher or lower than any other groups [168]. Copyright 2024, Elsevier Ltd.

It is difficult to control the balance between mechanical properties and bioactivity of bioresorbable PU to meet the comprehensive requirements of small-diameter vascular grafts. Li et al. [168] cross-linked the hard segment chain containing 2,2′-diene diethyl alcohol (SeDO) with diaminopyrimidine-covered polyethylene glycol (PEG) and ethylene glycol (EG), and designed the PU elastomers with different *M*w of PEG and mole ratios of diaminopyrimidine-capped PEG (DAPPEG)/diaminopyrimidine- capped EG (DAPEG) to achieve physiological elasticity of blood vessels, the modulus of PU synthesized with DAPPEG3k/DAPEG = 1/1 increased significantly, and the fracture strain and stress decreased significantly after immersion in water, and SeDO-based PU had less calcification due to the release of NO (Figure 10).

**Figure 11:**
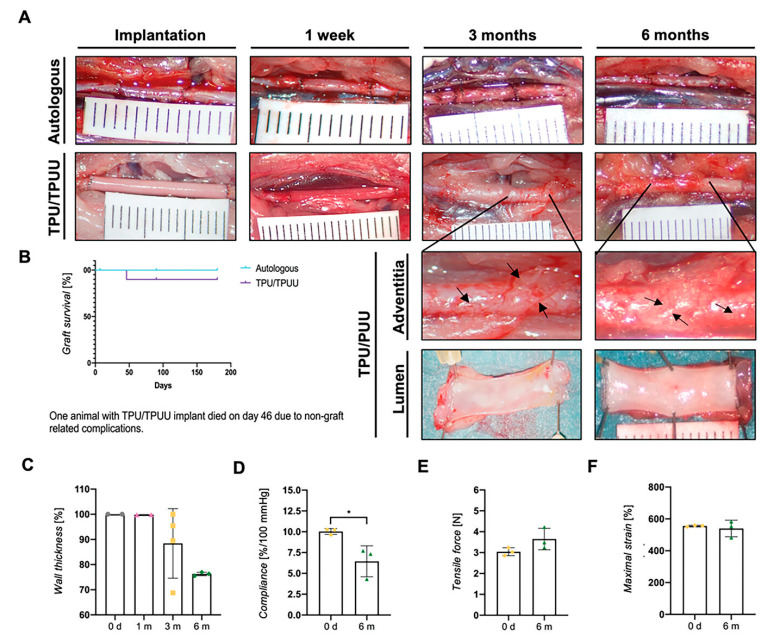
(**A**) The newly formed media layer of TPU/TPUU grafts after being implanted for 6 months in vivo, (**B**) the patency of TPU/TPUU grafts, (**C**) the wall thickness of TPU/TPUU grafts after 6 months of implantation, (**D**) the compliance of TPU/TPUU grafts after 6 months, (**E**,**F**) the tensile force and maximal applied strain values after 6 months of implantation. The results are presented as mean ± standard deviation, n = 3. Statistical analysis: t-test, *: *p* ≤ 0.05, mean ± SD. [169]. Copyright 2023, Wiley Ltd.

The mixed PU can be used to improve the mechanical properties of blood vessels. Rohringer et al. [169] prepared TPU using poly(tetrahydrofuran), HMDI, and Bis(2-hydroxyethyl) terephthalate (BHET). Poly(tetrahydrofuran), HMDI, BHET, and di(tert-butyl)ethylenediamine can be used to prepare a new type of self-strengthening polyurethane urea (TPUU), and the PU artificial blood vessels can be formed by using the co-mixed two materials. The biomechanical properties are sufficient even if the walls of artificial blood vessels become thin, and there was no inflammation, aneurysm, intimal hyperplasia, or thrombosis observed (Figure 11).

L-lysine, as a chain extender of biodegradable PU, can react with the isocyanate group to form a urea bond, which has stronger cohesion between the urea bond, making PU stronger, and further improves PU biocompatibility due to it being able to participate in the human physiological metabolism process without causing the body’s immune response [170]. Castillo-Cruz et al. [171] used PCL, HMDI, and lysine (Lys) as raw materials to synthesize segmented PU. Lys formed a crystal domain in the rigid segment of HMDI-Lys with HMDI, so that it had the potential of shape memory and high rupture strength, which can help regulate vascular graft cell behavior and surgical implantation. Zheng et al. [172] used PCL, PEG, and IPDI to form prepolymers, added ethyl lysine (Lys·OEt) as a chain extender for expansion, and then added gastrodin to form degradable PU materials, which improved the surface and mechanical properties of scaffoldings. Gastroetin can up-regulate the expression of vascular endothelial growth factor (VEGF), increase the binding to VEGF receptors on the endothelial surface, activate phosphodiesterase C-γ, promote the release of intracellular calcium ions, and cause the migration of endothelial cells and the formation of lumen. Thus the effective release of gastrodin in the PU matrix promoted vascular tissue regeneration. In addition, lysine can also be introduced into PU materials through the LDI pathway. LDI is non-toxic for degradation in vivo, and its hydrolyzed products are crucial to biological systems and have become a common isocyanate for biodegradable PU synthesis [165,173,174].

## 7. Summary and Prospect

There may be degradation of PU materials during the long-term implantation in vivo, resulting in a decline in the physical properties of artificial blood vessels, such as weak strength, cracks, etc., affecting normal use. In addition, PU materials can cause certain allergic reactions, leading to adverse reactions such as inflammation. Although a lot of research has been carried out in the field of PU materials for artificial blood vessels, there is still no successful commercial PU material through the verification of artificial blood vessel products. The performance of PU material is related to the structure and composition of the synthesized monomer, and different monomers have a great influence on its biostability and biocompatibility. Therefore, the stability and purity of the raw materials used in the production of PU need to be strictly controlled. In addition, the PU surface can also be functionalized to promote vascular endothelialization and increase its biocompatibility. However, how to implement this technology in the industrial production of PU remains to be further explored.

As described in this paper, PU is prone to hydrolysis and oxidative degradation in the blood environment after long-term implantation, and this degradation generally occurs in the soft segment of PU. The use of stable polyol compounds such as PHECD and PHC or the end-sealing treatment by PDMS and F compounds with low surface energy can stabilize the structure of the chain to reduce the degradation, and POSS nanoparticles filled in PU can reduce the porosity and increase the degree of crosslinking to improve stability. Many commercial PUs are a combination of the above ways to achieve long-term stability in vivo. Moreover, the biocompatibility of PU materials is also an issue that must be considered. Usually, antithrombotic or hydrophilic substances are grafted on their surface to reduce the occurrence of thrombosis, or functional substances are introduced into PU reactants to regulate the behavior of cells to promote the repair and regeneration of endothelial cells. In the future, functional modification methods can be applied to biostable PU, which will better promote the development of PU for artificial blood vessels. However, it is difficult to strike a balance between biocompatibility and the mechanical properties of bioresorbable PU due to the poor mechanical properties of its fast degradation. The mechanical properties can be improved through the design of heat-triggered and water-triggered shape memory materials to enhance the mechanical properties of materials, and the use of mixed materials and tyrosine-based chain extenders or isocyanates can also enhance the mechanical properties. Furthermore, the structure and proportion of each monomer of synthetic PU have a great influence on the mechanical properties, so it is necessary to reasonably regulate and achieve the ideal level. In short, it is necessary to reasonably adjust the structure of PU to achieve the balance of biostability, biocompatibility, and mechanical properties to realize the clinical application of PU artificial blood vessels, and its degradation properties and mechanical properties are highly correlated.

Each component of synthetic polyurethane is the key to affecting the structure and properties of PU. To promote the development of PU for artificial blood vessels as soon as possible, it is necessary to further develop stable polyols and functional additives to improve the long-term stability and biocompatibility of PU in vivo. Although the PU materials for PU scaffolds do not require long-term stability, regulating the balance between mechanical loss due to degradation and endothelialization rate is still the key to current research. In the continuous exploration of new PU materials, the mixture of different types of PU may be able to achieve performance adjustment. At present, many researchers are developing non-cyanate polyurethane (NCPU) to reduce the toxicity of PU. However, its production efficiency and molecular weight are difficult to control. A few studies have applied NCPU in prosthetic heart valves and medical adhesives, but they have yet to be applied in artificial blood vessels. In addition, the use of various monomers to design functional PU materials, such as PU with shape memory and self-reinforcing properties according to vascular application scenarios, still needs to be developed further.

## Figures and Tables

**Table 1:** The main structure of PU monomer and its influence on properties.

Monomer	Key Chemical Structure	Influence of Properties
Polyols	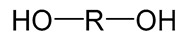	Biostability, elasticity properties, and biocompatibility
Isocyanates	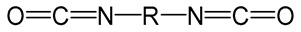	Reaction kinetics, mechanical strength, and biocompatibility
Chain extender	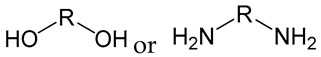	Mechanical strength

**Table 2:** The common polyol compounds for the synthesis of PU.

Type	Chemical Name	Chemical Structure
Polyester	Poly(ε-caprolactone) diol (PCL)	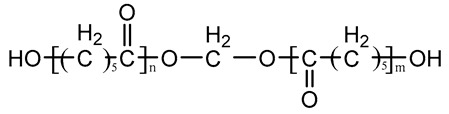
Polyether	Poly(ethylene glycol) (PEG)	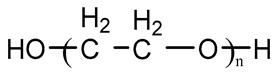
	Poly(tetramethylene oxid) diol (PTMO)	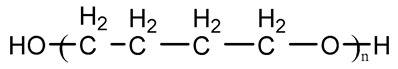
	Poly(hexamethylene oxid) diol (PHMO)	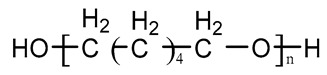
Polycarbonate	Poly(1,6-hexyl-1,2- ethylcarbonate) diol (PHECD)	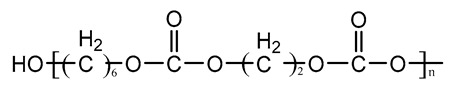
	Poly(hexamethylene carbonate) diol (PHC)	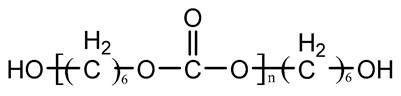
Silicone	Polydimethylsiloxane (PDMS)	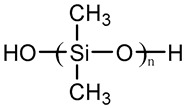

**Table 3:** The common isocyanate compounds for synthesizing PU.

Type	Chemical Name	Chemical Structure
Aromatic	4,4’-methylene bisphenyl diisocyanate (MDI)	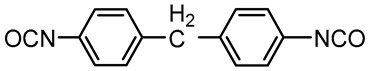
	Toluene diisocyanate (TDI)	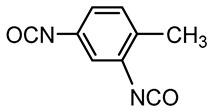
Aliphatic	Hydrogenated 4,4’-methylene bisphenyl diisocyanate (H_12_MDI)	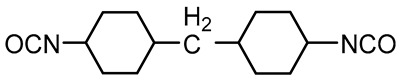
	Hexane diisocyanate (HDI)	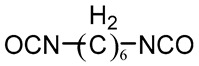
	Isophorone diisocyanate (IPDI)	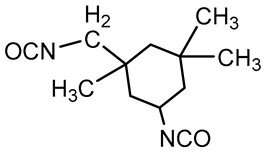
	L-Lysine ethyl ester diisocyanate (LDI)	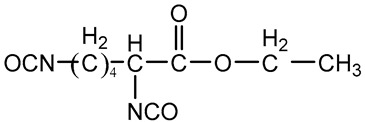

**Table 4:** The common chain extenders for the synthesis of PU.

Type	Chemical Name	Chemical Structure
Glycol	1,4-Butanediol (BDO)	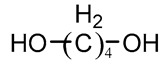
	Ethylene glycol (EG)	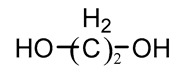
	1,6-Hexanediol (HDO)	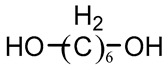
Diamine	Ethylene diamine (ED)	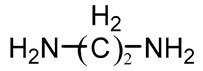
Amino alcohol	Lysine	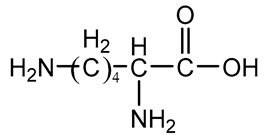

**Table 5:** The typical biomedical commercial PU products.

Type	Name	Component	Company	Advantages and Disadvantages	Refs.
PEtUs	Biomer^®^	MDI/ED/PTMH	Ethicon, New Jersey, NJ, USA	Difficult to process	[80]
	Pellethane 2363 series	MDI/BDO/PTMO	Lubrizol, Wickliffe, OH, USA	Oxidative degradation	[89]
	Tecoflex^®^	HMDI/BDO/PTMO	Lubrizol, Wickliffe, OH, USA	Faster degradation than Pellethane	[90]
PDMS modified PEtUs	Biospan^®^ S	MDI/PTMO/ED and 1,3-CHD, the end of the chain is modified by PDMS	DSM, Maastricht, The Netherlands	Long term stability	[91]
	Pursil^®^	MDI/PHMO/BDO, the end of the chain is modified by PDMS	DSM, Maastricht, The Netherlands	Long term stability	[85]
F modified PEtUs	Biospan^®^ F	MDI/PTMO/ED and 1,3-CHD, the end of the chain is modified by fluorocarbons	DSM, Maastricht, The Netherlands	Long term stability	[91]
PCU	Bionate^®^	MDI/BDO(ED)/PHECD	DSM, Maastricht, The Netherlands	Long term stability	[92]
	Carbothane^®^	MDI(HMDI)/BDO(ED)/PHECD	Lubrizol, Wickliffe, OH, USA	Long term stability	[88]
Si modified-PCU	CarboSil^®^	MDI/BDO(ED)/PHECD, the end of the chain is modified with silicone	DSM, Maastricht, The Netherlands	Long term stability	[93]

## Data Availability

Not applicable.
